# Association of Medicaid Expansion Under the Patient Protection and Affordable Care Act With Use of Long-term Care

**DOI:** 10.1001/jamanetworkopen.2020.18728

**Published:** 2020-10-01

**Authors:** Courtney Harold Van Houtven, Brian E. McGarry, Eric Jutkowitz, David C. Grabowski

**Affiliations:** 1 Center of Innovation to Accelerate Discovery and Practice Transformation, Durham Veterans Affairs Health Care System, Durham, North Carolina; 2 Department of Population Health Sciences, Duke University, Durham, North Carolina; 3 Duke-Margolis Center for Health Policy, Duke University, Durham, North Carolina; 4 Division of Geriatrics & Aging, Department of Medicine, University of Rochester, Rochester, New York; 5 Department of Health Services, Policy, and Practice, Brown University, Providence, Rhode Island; 6 Department of Health Care Policy, Harvard Medical School, Boston, Massachusetts

## Abstract

**Importance:**

Medicaid expansion is associated with increased access to health services, increased quality of medical care delivered, and reduced mortality, but little is known about its association with use of long-term care.

**Objective:**

To examine the association of Medicaid expansion under the Patient Protection and Affordable Care Act (ACA) with long-term care use among newly eligible low-income adults and among older adults whose eligibility did not change.

**Design, Setting, and Participants:**

This difference-in-difference cohort study used data from the Health and Retirement Study, a nationally representative longitudinal survey of persons 50 years or older. Long-term care use from 2008 to 2012 was compared with use from 2014 to 2016 among low-income adults aged 50 to 64 years without Medicare coverage residing in states in which Medicaid coverage expanded in 2014 and those living in states without expansion. Low-income adults who were covered by Medicare and were ineligible for expanded Medicaid were also included in the analysis. Data were analyzed from January 15, 2018, to December 31, 2019.

**Exposures:**

Residence in a state with Medicaid expansion in 2014.

**Main Outcomes and Measures:**

Any home health care use or any nursing home use in 2014 or 2016. All estimates are weighted to account for the Health and Retirement Study sampling design.

**Results:**

Among the 891 individuals likely eligible for expanded Medicaid, the mean (SD) age was 55.2 (3.1) years; 534 (53.4%) were women, 482 (49.5%) were married, and 661 (45.9%) were White non-Hispanic. Before the ACA-funded Medicaid expansion, 0.4% (95% CI, −0.3% to 1.1%) in expansion states and 1.0% (95% CI, −0.1% to 2.2%) in nonexpansion states used nursing homes, and 1.9% (95% CI, 0.4%-3.4%) in expansion states and 7.1% (95% CI, 4.7%-9.5%) in nonexpansion states used any formal home care. The ACA-funded Medicaid expansion was associated with an increase of 4.4 percentage points (95% CI, 2.8-6.1 percentage points) in the probability of any long-term care use among low-income, middle-aged adults, with increases in home health use (3.8 percentage points; 95% CI, 2.0-5.6 percentage points) and in any nursing home use (2.1 percentage points; 95% CI, 0.9-3.3 percentage points).

**Conclusions and Relevance:**

In this study, ACA-funded Medicaid expansion was associated with an increase in any long-term care use among newly eligible low-income, middle-aged adults, suggesting that the population covered by the Medicaid expansion may have had unmet long-term care needs before expansion.

## Key Points

**Question:**

Was there an association of the Patient Protection and Affordable Care Act (ACA) Medicaid expansion with long-term care use among newly eligible low-income adults and older adults whose eligibility did not change?

**Findings:**

In this cohort study of Health and Retirement Study data for 891 individuals from 2008 to 2016, ACA-funded Medicaid expansion was associated with a statistically significant increase in the probability of any long-term care use among low-income, middle-aged adults.

**Meaning:**

In this study, Medicaid expansion funded by the ACA was associated with increased access to long-term care for newly eligible low-income, middle-aged adults, suggesting that the population covered by the Medicaid expansion may have had unmet long-term care needs before expansion.

## Teaser

This cohort study uses data from the Health and Retirement Study to examine the association between Patient Protection and Affordable Care Act funded–Medicaid expansion in 2014 and long-term care use among low-income, middle-aged adults newly eligible for Medicaid.

## Introduction

The Patient Protection and Affordable Care Act (ACA) increased health insurance coverage for 20 million individuals in the US, with much of this gain occurring through expansion of the state-administered Medicaid program.^1^ Although Medicaid expansion has been shown to be associated with increased access to health services, increased quality of medical care delivered, and reduced mortality,^2,3,4,5^ little is known about its association with individuals’ use of long-term care (LTC), including use of formal home health or nursing home care. This association is important because Medicaid finances 50% of all LTC in the US,^6^ and nearly two-thirds of all Medicaid spending focuses on older adults and adults with disability.^7^ Furthermore, persons with disability prefer to remain in the least restrictive setting and have the right to do so by law (eg, Olmstead decision)^8,9,10,11^; thus, it is important to understand how any increased access to care affects home care vs nursing home care.

The populations most likely to use Medicaid-funded LTC were exempted from ACA Medicaid expansion. Adults 65 years or older or those younger than 65 years but entitled to Medicare coverage because of disability were ineligible for expanded Medicaid.^12^ As such, Medicaid expansion may have been associated with increased access to LTC for young to middle-aged adults and for adults who had no disability or a new or temporary disability. Because of spillovers, expansion might also have been indirectly associated with increased LTC access for older adults or adults with disability. Spillovers may arise from the so-called welcome mat phenomenon, in which, for example, increased publicity about Medicaid and a streamlined application process attract people who were eligible under traditional criteria but who had not enrolled previously and then underwent the eligibility process.^13,14^ Other structural changes to the Medicaid program after ACA expansion that targeted potential eligible adults might also have been associated with increased coverage and with increased demand for LTC. For example, the increase in case management as a part of contracts for accountable care organizations could make case managers more aware of Medicaid application processes. There also might have been increased LTC use among existing Medicaid beneficiaries after the ACA expansion owing to increased funding for Medicaid and to persistent unmet needs among older adults and persons with disability.

This study used state-level variation in the implementation of ACA-funded Medicaid expansion to assess for the first time, to our knowledge, its association with any home health care use (either skilled or unskilled) or nursing home use for any cause, referred to as *any formal LTC*. The direct association of ACA-funded Medicaid expansion with LTC use was assessed by examining individuals likely to gain Medicaid coverage through the ACA, including adults with household incomes less than 138% of the federal poverty level (FPL) and without Medicare coverage. The association of the spillover policy with LTC use was also assessed for individuals 65 years or older and middle-aged adults with Medicare coverage.

First, we evaluated whether individuals most likely to gain Medicaid eligibility after ACA Medicaid expansion experienced increased use of LTC. The hypothesis was that ACA Medicaid expansion was associated with increased LTC use among low-income, middle-aged adults who directly gained access from expansion. Second, we evaluated whether there were spillovers of ACA Medicaid expansion. The presence of spillovers was tested by examining whether individuals previously ineligible for Medicaid through the ACA, including older adults or adults with disability who were covered by Medicare, experienced increased use of LTC in Medicaid expansion states compared with nonexpansion states. The hypothesis was that any increased LTC use among older adults or adults with disability who had Medicare suggested that there were spillovers of the policy that were indirectly associated with increased LTC use.

## Methods

This cohort study used data from the Health and Retirement Study (HRS) from 2008 to 2016. The HRS is a nationally representative, publicly available, biannual survey of middle-aged and older adults in the US. It contains detailed income and asset information for identifying individuals likely to be affected by ACA Medicaid expansions. It also includes information about insurance coverage, health status, and use of health services including LTC. Publicly available data were linked with restricted information about respondents’ state of residence in each survey wave to determine respondents’ exposure to Medicaid expansion. This research complied with the accepted institutional review board protocol of Harvard University. The Duke University institutional review board deemed the study exempt because the data were deidentified. This study followed the Strengthening the Reporting of Observational Studies in Epidemiology (STROBE) reporting guideline.

The study sample included individuals with HRS data from 2008 to 2016. To be eligible, respondents had to be 50 years or older and residing in the community in at least 1 study wave. Respondents who changed their state of residence from 2012 to 2016 were excluded. To test the direct association of ACA-funded Medicaid expansion with LTC use, the sample was restricted to individuals who did not have Medicare coverage in any of the survey waves from 2008 to 2014 and whose household income was less than 138% of the FPL in 2014. To test for spillovers, the sample was limited to those with Medicare coverage (owing to disability or age) in 2014 and with a household income less than 138% of the FPL in 2014. These individuals would not be expected to experience a change in Medicaid eligibility based on ACA-funded expansion rules.

### Definition of Treatment

To identify a set of states that were and were not exposed to Medicaid expansion, the comparison focused on states that expanded Medicaid in 2014 (20 states) and states that did not expand Medicaid at any point during the study period (19 states). The final sample consisted of 33 states owing to HRS data coverage. The respondents’ state of residence was used to classify individuals in a treatment group (lived in a Medicaid expansion state) and a comparison group (lived in a state that did not expand its Medicaid program).

### Definition of Outcomes

Home health care use was measured using responses to the following question: “[Since previous wave interview month/year] / [In the past 2 years], has any medically trained person come to your home to help you, yourself?” In addition, in the HRS helper-level file, respondents reported whether they received any assistance with each of 6 activities of daily living (walking, dressing, bathing, eating, getting in and out of bed, and using the toilet) and 6 instrumental activities of daily living (preparing hot meals, shopping for groceries, making telephone calls, taking medications, managing money, and driving). For those who indicated yes, this assistance was coded as home health care use when it came from an organization, an employee of an institution, a paid helper, or a health professional.

Nursing home use was defined using responses to the following question: “[Since previous wave interview month/year] / [In the past 2 years], have you been a patient overnight in a nursing home, convalescent home, or other long-term health care facility?” Respondents who answered yes were considered to have used a nursing home in the corresponding survey wave. Respondents were categorized as having received any LTC if they reported using either nursing home care or home health care.

### Statistical Analysis

Data analysis was performed from January 15, 2018, to December 31, 2019. State-level variation in the decision to implement ACA-funded Medicaid expansion was used to estimate direct and spillover associations with LTC use. The difference-in-difference study design compared LTC use from 2008 to 2012 with LTC use from 2014 to 2016 among individuals residing in states that expanded Medicaid coverage compared with individuals residing in states that did not expand Medicaid coverage. The primary independent variable of interest in the linear regression models was an interaction term between an indicator for living in an expansion state and an indicator for postexpansion waves (2014 and 2016) (eAppendix 1 in the Supplement gives model specification details). All models were adjusted for age, sex, race/ethnicity, marital status, educational attainment, income, assets, labor market status, self-reported health (fair or poor, with excellent, very good, or good as the reference), number of chronic conditions reported, number of limitations to activities of daily living (eg, bathing), number of limitations to instrumental activities of daily living (eg, cooking), and whether a proxy respondent helped the HRS respondent complete the survey. Complete case analysis was performed using Stata, version 15 (StataCorp). All estimates are weighted to account for the HRS sampling design.

Separate models were estimated for the 3 main outcomes of interest: any home health care use, any all-cause nursing home use, and any formal LTC use. State-level fixed effects were used in all models to control for state-level differences that may have affected LTC markets and use. Standard errors were clustered at the state level.

The primary regression model focused on respondents likely to gain Medicaid coverage through the ACA (those with household income <138% of the FPL and not enrolled in Medicare). Spillover estimates (ie, those owing to the existence of the welcome mat phenomenon or other reasons, such as expanded Medicaid budgets globally) were obtained by applying the same model to respondents with Medicare coverage (owing to disability or age qualification) and low income (<138% of the FPL).

Several sensitivity analyses were used to test the robustness of our results. Specifically, the difference-in-difference models were reestimated using person-level rather than state-level fixed effects to better control for unobserved individual-level differences within the sample. Person-level random effects were also used. Trends in LTC use among middle-aged, low-income adults were also compared with those among middle-aged, higher-income adults living within the same expansion state. Adults with higher income (household income >150% of the FPL) should not have been affected by ACA Medicaid expansions; therefore, they served as an alternate comparison group that was drawn from within expansion states.

## Results

Among the 891 individuals likely eligible for expanded Medicaid, the mean (SD) age at study entry was 55.2 (3.1) years; 534 (53.4%) were women, 482 (49.5%) were married, and 661 (45.9%) were White non-Hispanic. Among the middle-aged, lower-income cohort residing in expansion states (341 [38.3%]; 1318 person-years), the mean (SD) age was 56.6 (3.6) years; 52.7% (95% CI, 45.0%-60.2%) were women, 38.5% (95% CI, 32.1%-45.2%) were married, 49.9% (95% CI, 40.1%-59.7%) were White non-Hispanic, 22.2% (95% CI, 17.1%-28.3%) had less than a high school education, 22.5% (95% CI, 17.2%-28.8%) reported being retired, and the mean (SD) number of chronic conditions was 1.7 (1.5) before ACA Medicaid expansion (Table 1). Individuals in nonexpansion states had lower educational attainment and higher rates of marriage and retirement than individuals in expansion states (Table 1).

**Table 1.** Sample Characteristics and Formal Long-term Care Use Before Medicaid Expansion Under the ACA in 2014^**a**^

Characteristic	Individuals likely eligible for Medicaid expansion^b^		Individuals not eligible for ACA Medicaid expansion^c^	
	Nonexpansion state (n = 550)	Expansion state (n = 341)	Nonexpansion state (n = 959)	Expansion state (n = 512)
Person-years^d^	2197	1318	4117	2246
Age, mean (SD), y	56.4 (3.9)	56.6 (3.6)	69.2 (10.9)	70.1 (10.6)
Female	55.0 (48.0 to 61.9)	52.7 (45.0 to 60.2)	69.5 (64.7 to 73.8)	71.7 (66.5 to 76.3)
White, non-Hispanic	47.4 (31.7 to 63.7)	49.9 (40.1 to 59.7)	44.9 (37.5 to 52.5)	61.6 (53.4 to 69.1)
Less than high school education	38.4 (28.3 to 49.6)	22.2 (17.1 to 28.3)	49.9 (43.3 to 56.4)	35.5 (29.2 to 42.3)
Bachelor’s degree or higher	11.6 (7.6 to 17.3)	20.7 (14.8 to 28.1)	6.9 (4.2 to 11.1)	9.8 (6.7 to 14.3)
Married	50.8 (42.8 to 58.8)	38.5 (32.1 to 45.2)	33.7 (28.8 to 39.0)	25.8 (21.6 to 30.6)
Retired	31.3 (27.5 to 35.4)	22.5 (17.2 to 28.8)	75.9 (72.4 to 79.0)	80.7 (77.0 to 83.9)
Annual household income, median (IQR), $	14 496 (6624 to 30 000)	17 400 (7500 to 36 001)	12 714 (9120 to 21 480)	13 200 (9760 to 21 040)
Assets, median (range), $	20 000	18 010	36 900	17 500
Proxy respondent	0.9 (0.5 to 1.5)	2.8 (0.7 to 10.4)	3.1 (2.0 to 4.8)	3.1 (1.6 to 5.9)
Fair to poor self-reported health	47.1 (37.8 to 56.6)	36.7 (28.7 to 45.5)	50.6 (47.2 to 54.0)	48.1 (42.6 to 53.6)
Limitations, mean (SD), No.				
Activities of daily living	0.4 (1.1)	0.3 (0.8)	0.7 (1.4)	0.5 (1.1)
Instrumental activities of daily living	0.3 (0.9)	0.2 (0.7)	0.6 (1.2)	0.4 (1.0)
Chronic conditions, mean (SD), No.	1.8 (1.7)	1.7 (1.5)	2.6 (1.7)	2.5 (1.5)
Annual long-term care use, mean (95% CI), %				
Any formal long-term care use	7.0 (2.9 to 11.2)	2.3 (−1.4 to 6.0)	13.7 (11.0 to 16.5)	13.4 (10.0 to 16.8)
Home care use	7.1 (4.7 to 9.5)	1.9 (0.4 to 3.4)	13.2 (10.3 to 16.1)	12.5 (8.9 to 16.1)
Nursing home use	1.0 (−0.1 to 2.2)	0.4 (−0.3 to 1.1)	2.4 (1.5 to 3.3)	4 (2.4 to 5.5)

Abbreviations: ACA, Patient Protection and Affordable Care Act; IQR, interquartile range.

^a^ Data are presented as percentage (95% CI) of individuals unless otherwise indicated and are from 2008 to 2012. All estimates are weighted to account for the Health and Retirement Study sampling design. Missingness was 5% or less for each covariate.

^b^ Respondents with a household income less than 138% of the federal poverty level and without Medicare coverage in 2014.

^c^ Respondents with a household income less than 138% of the federal poverty level and with Medicare coverage in 2014.

^d^ From 2008 to 2016.

In the spillover cohort of Medicare beneficiaries, expansion state respondents (n = 512; 2246 person-years) had a mean (SD) age of 70.1 (10.6) years; 71.7% (95% CI, 66.5%-76.3%) were women, 61.6% (95% CI, 53.4%-69.1%) were White non-Hispanic, 80.7% (95% CI, 77.0%-83.9%) reported being retired, and the mean (SD) number of chronic conditions was 2.5 (1.5) before ACA Medicaid expansion. Individuals in the spillover cohort in nonexpansion states were less likely to be White non-Hispanic, had lower educational attainment, and were less likely to be retired compared with those in expansion states.

Among individuals likely eligible for ACA-expanded Medicaid, LTC use was low before Medicaid expansion (Table 1). In expansion states, from 2008 to 2012, 2.3% (95% CI, −1.4% to 6.0$) of individuals reported any formal LTC use, with 1.9% (95% CI, 0.4%-3.4%) reporting home care use and 0.4% (95% CI, −0.3% to 1.1%) reporting at least 1 nursing home stay. In nonexpansion states, 7.0% (95% CI, 2.9%-11.2%) reported any formal LTC use (7.1% [95% CI, 4.7%-9.5%] and 1.0% [95% CI, −0.1% to 2.2%] used home care and nursing home care, respectively; some individuals used both). Of the adults ineligible for expanded Medicaid, 13.7% (95% CI, 11.0%-16.5%) in nonexpansion states and 13.4% (95% CI, 10.0%-16.8%) in expansion states reported any formal LTC use.

**Figure 1.**
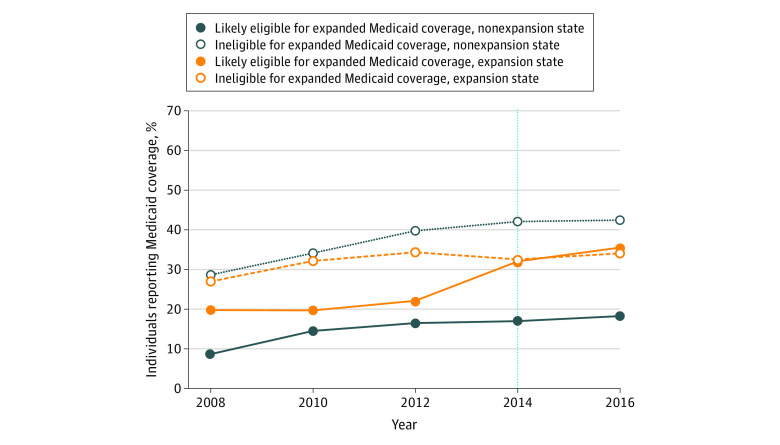
Self-reported Medicaid Coverage by Patient Protection and Affordable Care Act Medicaid Expansion Status of a Respondent’s State Estimates were weighted to account for the Health and Retirement Study sample design. The vertical dashed line represents the year of Medicaid expansion under the Patient Protection and Affordable Care Act.

There was a substantial increase in Medicaid insurance enrollment from 2008 to 2016 among individuals living in states that expanded Medicaid in 2014, with an increase from 19.7% in 2008 to 35.5% in 2016 among the population most likely to directly gain coverage after expansion (those aged 50 to 64 years whose household income was <138% of the FPL) (Figure 1). By contrast, among individuals residing in nonexpansion states who were most likely to directly gain coverage, Medicaid enrollment increased from 8.6% to 18.2% during the same period .

**Figure 2.**
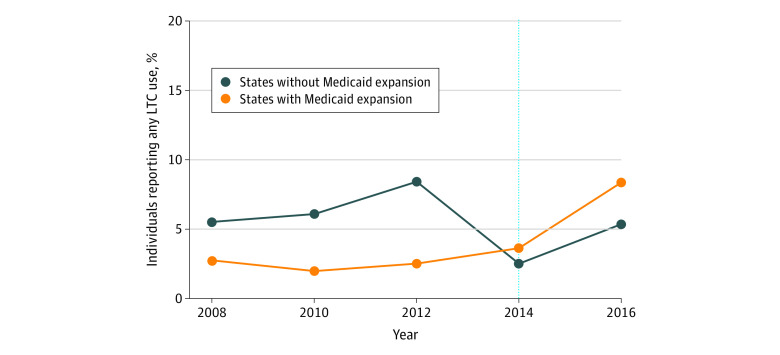
Self-reported Use of Any Formal Long-term Care (LTC) by Patient Protection and Affordable Care Act Medicaid Expansion Status of a Respondent’s State Respondents were individuals with household incomes less than 138% of the federal poverty level and without Medicare coverage. Estimates were weighted to account for the Health and Retirement Study sample design. The vertical dashed line represents the year of Medicaid expansion under the Patient Protection and Affordable Care Act.

Data indicating the estimated direct association of ACA Medicaid expansion with changes in use of formal LTC, any formal home health care, and any nursing home care appear in Table 2. For the population expected to gain eligibility (lower income, <65 years of age, and not enrolled in Medicare), adjusted estimates showed that ACA-funded Medicaid expansion was associated with an increase of 4.4 percentage points (95% CI, 2.8-6.1 percentage points) in any formal LTC use, representing a nearly 2-fold increase from the preexpansion use rate of 2.3% (Figure 2). Specifically, Medicaid expansion was associated with increases of 3.8 percentage points (95% CI, 2.0-5.6 percentage points) in the use of any home health care and 2.1 percentage points (95% CI, 0.9-3.3 percentage points) in any nursing home use. Tests for parallel trends in the outcomes before 2014 between expansion and nonexpansion states supported our analytic approach (eAppendix 2 and eTable 1 in the Supplement). Results were robust to the use of an alternate, within-state comparison group as well as person-level fixed and random effects (eAppendix 3 and eTable 2 in the Supplement). Among the population of respondents not expected to be directly affected by ACA-funded Medicaid expansion (those with Medicare coverage before expansion), these estimates provide no evidence that spillovers were associated with changes in formal LTC use (Table 2).

**Table 2.** Differential Change in Self-reported Long-term Care Use After Medicaid Expansion Under the Patient Protection and Affordable Care Act in 2014^**a**^

Type of long-term care	Change in long-term care use in expansion vs nonexpansion states after expansion, percentage points (95% CI)^b^	
	Likely eligible for Medicaid expansion^c^	Not eligible for expanded Medicaid^d^
Home health care	3.8 (2.0 to 5.6)	1.7 (−2.5 to 5.8)
Nursing home care	2.1 (0.9 to 3.3)	−1.1 (−3.7 to 1.5)
Any formal long-term care	4.4 (2.8 to 6.1)	1.7 (−2.7 to 6.1)

^a^ Estimates were obtained from linear regression models that controlled for age, sex, race/ethnicity, marital status, educational attainment, income, assets, labor market status, self-reported health, chronic condition count, a count of activity of daily living limitations, a count of instrumental activity of daily living limitations, and use of a proxy respondent. Models also included year and state fixed effects. Separate models were estimates for each outcome within each study sample.

^b^ Obtained from robust SEs clustered at the state level.

^c^ Respondents with a household income less than 138% of the federal poverty level and without Medicare coverage in 2014.

^d^ Respondents with a household income less than 138% of the federal poverty level and with Medicare coverage in 2014.

## Discussion

The ACA has been shown to be associated with increased health care access and use, improved health outcomes, and reduced mortality.^2,4,15^ Recent studies have examined spillovers to nonhealth sectors and have found associated reductions in eviction rates^16^ and no changes in Social Security income or Social Security disability income applications.^17^ To our knowledge, this study provides the first evidence that ACA-funded Medicaid expansion was directly associated with increased formal LTC use among the population that benefited directly from expansion (nonelderly adults with low income). This associated increase occurred in both greater formal home health care use and greater nursing home use.

This finding was likely attributable to the increased access to LTC through insurance rather than just a substitution of formal services for unpaid family care. Of importance, this interpretation was supported by a supplemental analysis that found no evidence of a reduction in the number of middle-aged, lower-income adults receiving informal care (defined as assistance with activities of daily living from family or unpaid friends) after the ACA Medicaid expansion (eAppendix 4 and eTable 3 in the Supplement). Thus, the Medicaid expansion appears to have been associated with a net gain in LTC access for low-income, middle-aged adults in the US (ie, use of formal care increased without a reduction in receipt of informal care). This finding also supports the observation that the population newly eligible for Medicaid after expansion may have had unmet LTC needs before expansion.^18^

Medicaid coverage expansion was associated with a larger increase in home-based service use than in nursing home use. The increase in use of home health fits with the conventional wisdom that individuals would generally prefer to receive LTC services in the least-restrictive setting possible. These findings are also consistent with prior research indicating that individuals are responsive to more generous public home-care benefits.^19^ The literature on the response to more generous Medicaid benefits for nursing home services is more nuanced. In the aggregate, nursing home use historically has not been found to be associated with Medicaid coverage expansions among older adults in the general population.^20^ This fits with the idea that individuals may not want to enter a nursing home if they are able to receive services elsewhere. However, in research isolating the lowest-income, oldest, and least healthy older adults, more generous Medicaid coverage has been found to be associated with an increase in nursing home use.^21^ Thus, there are heterogeneous responses to Medicaid expansion for high-risk subgroups of older adults. Given the analyses in this study focused on the expansion of Medicaid among individuals with a household income less than 138% of the FPL, the current findings support the conclusions of that earlier research.

In addition, this study did not find an association between ACA-funded Medicaid expansion and the use of formal LTC among Medicare beneficiaries (eligible because of age or disability). This result is intuitive because the ACA did not expand Medicaid coverage for individuals receiving Medicare. However, owing to the newness of expansion, there may have been an association between coverage expansion and coverage persistence that was not observed. For example, earlier research found a dynamic welcome-mat effect in which Medicaid expansion through the ACA was associated with a 9.5% increase in the likelihood that individuals with low income would retain their Medicaid benefit after they turned 65 years of age and became eligible to be dually enrolled in Medicaid and Medicare.^22^ With a larger data set and additional years of follow-up, future research might examine whether this could have positive spillovers to access and use of LTC among older adults.

### Limitations

This study has limitations. The HRS sample is relatively small when limited to the populations of interest (middle-aged, low-income individuals without Medicare coverage and older adults with low income and Medicare coverage). Adults younger than 50 years may also experience changes in LTC use but are not included in the HRS sampling frame other than through marriage to a respondent 50 years or older. In addition, this analysis was limited to 33 states and therefore may not be representative of the entire US. It is not possible to discern intensity or episode length of home health care using data from the HRS. In addition, other researchers have documented the importance of linking Medicare-claims data to HRS data to accurately measure nursing home care.^14,23^ In particular, recent evidence indicates that stays in skilled-nursing facilities are undercounted in the HRS data.^23^ Thus, our estimates of nursing home use may more accurately reflect long-term (mainly Medicaid-covered) stays. Assuming that any undercounting occurred randomly and regardless of whether a person lived in an expansion state and that undercounting did not change over time, our nursing home outcome measure would represent an underestimate of total nursing home use but not a biased estimate. It is difficult to discern short vs long nursing home stays using HRS data alone. In addition, our results could have combined ACA Medicaid expansion with other unobserved policy feedback loops.^24^ To minimize bias from the possibility of unobserved policy feedback loops, the models included time and state-level fixed effects.

## Conclusions

In this cohort study, ACA-funded Medicaid expansion was directly associated with increases in formal LTC use among the population expected to benefit from expansion. Future research is needed to determine the effects of this increased use on beneficiaries’ health and social outcomes.

## Notes

Supplement. **eAppendix 1.** Regression Specification **eAppendix 2.** Parallel Trends Test **eAppendix 3.** Sensitivity Analyses **eAppendix 4.** Informal Care Use **eTable 1.** Results of Parallel Trends Testing for Formal LTC Use Outcomes **eTable 2.** Results of Sensitivity Analyses **eTable 3.** Effect of ACA-funded Medicaid Expansion on Informal Long-term Care Use
