# Supplementary material for: Association of Medicaid Expansion Under the Patient Protection and Affordable Care Act With Use of Long-term Care
Source: JAMA Netw Open. 2020 Oct 1;3(10):e2018728. doi: 10.1001/jamanetworkopen.2020.18728 (PMC7530637; doi:10.1001/jamanetworkopen.2020.18728)
Supplement: Supplement. — eAppendix 1. Regression Specification eAppendix 2. Parallel Trends Test eAppendix 3. Sensitivity Analyses eAppendix 4. Informal Care Use eTable 1. Results of Parallel Trends Testing for Formal LTC Use Outcomes eTable 2. Results of Sensitivity Analyses eTable 3. Effect of ACA-funded Medicaid Expansion on Informal Long-term Care Use [file jamanetwopen-e2018728-s001.pdf]

Supplementary Online Content

Van Houtven CH, McGarry BE, Jutkowitz E, Grabowski DC. Association of Medicaid expansion under the Patient Protection and Affordable Care Act with use of long-term care. *JAMA Netw Open*. 2020;3(10):e2018728.
doi:10.1001/jamanetworkopen.2020.18728

eAppendix 1. Regression Specification
eAppendix 2. Parallel Trends Test
eAppendix 3. Sensitivity Analyses
eAppendix 4. Informal Care Use
eTable 1. Results of Parallel Trends Testing for Formal LTC Use Outcomes
eTable 2. Results of Sensitivity Analyses
eTable 3. Effect of ACA-funded Medicaid Expansion on Informal Long-term Care Use

This supplementary material has been provided by the authors to give readers additional information about their work.

© 2020 Van Houtven et al. *JAMA Network Open*.

Supplemental Materials for:

Association of Medicaid Expansion Under the Affordable Care Act with Use of Long Term Care

© 2020 Van Houtven et al. *JAMA Network Open*.

eAppendix 1. Regression Specification

To estimate the impact of ACA-funded Medicaid expansion on long-term care (LTC) use, we utilize a difference-in-difference approach. Specifically, we compare long-term care use among individuals living in states that expanded their Medicaid program before and after expansion (i.e., 2014) relative to the change long-term care use experienced by comparable individuals living in non-expansion states over the same time period. This approach is summarized in Equation S1 below.

$$Y_{i,s,t} = T_s * Post_t + X_i + \gamma + \delta + \varepsilon_{i,s,t} \qquad \text{S1}$$

Where $Y_{i,s,t}$ is an indicator for whether individual i in state s and year t reports any of the LTC use outcomes of interest. X_i represents a vector on individual-level controls described in the manuscript, γ is a state fixed effect that captures baseline differences in LTC use among states, and δ is a year fixed effect that captures time trends. T_s is an indicator for state s being a 2014 expansion state and $Post_t$ is an indicator years following ACA-Medicaid expansion (i.e., 2014-2016). The interaction between $T_s * Post_t$ represents the effect estimate of interest (i.e., the difference-in-difference estimator) which captures the relative change in LTC between individuals in expansion states and non-expansion states before and after Medicaid expansion. $\varepsilon_{i,s,t}$ is a robust standard error terms clustered at the state level.

eAppendix 2. Parallel Trends Test

A key assumption of the difference-in-differences approach is that trends in the outcome are parallel prior to the intervention between the treatment and control groups. We test

this assumption by examining outcomes over the pre-Medicaid expansion study year (2008-2012) using a linear regression with an interaction term between a continuous time variable and an indicator for living in an expansion state. Results are presented in Table S1. The null results do not support the existence of significant differential trends in long-term care use in the pre-period between expansion and non-expansion states.

eTable 1. Results of Parallel Trends Testing for Formal LTC Use Outcomes

	Direct Population	Indirect Population
	Test for differential pre-period trends between expansion and non-expansion states (pp)	Test for differential pre-period trends between expansion and non-expansion states (pp)
	(95% Confidence Interval)	*(95% Confidence Interval)*
Home Care Use	-0.40	1.32
	(-2.01, 1.21)	*(-1.42, 4.05)*
Nursing Home Use	-0.70	0.72
	(-1.72, 0.33)	*(-0.71, 2.14)*
Formal long-term care use	-0.81	1.50
	(-2.36, 0.75)	*(-1.63, 4.64)*

© 2020 Van Houtven et al. *JAMA Network Open*.

eAppendix 3. Sensitivity Analyses

To check the robustness of our results, we perform a series of sensitivity analyses (see Table S2). Preferred results presented in the main manuscript appears in columns (I) of Table S2. Column (II) displays difference-in-difference estimates from models that used person-level fixed effects instead of state fixed effects. Column (III) contains results from models with person-level random effects. For the sample of respondents likely eligible for ACA-expanded Medicaid, column (IV) displays results from a comparison with an alternate control group. Specifically, we compared individuals likely eligible for Medicaid expansion to individuals without Medicare coverage living in the same state who were ineligible for Medicaid due to their household income (i.e., income >150% of the federal poverty level). Results are robust to these alternate specifications.

© 2020 Van Houtven et al. *JAMA Network Open*.

eTable 2. Results of Sensitivity Analyses

	Likely Eligible for ACA-Medicaid Expansion				Not Eligible for ACA-Expanded Medicaid		
	(I)	(II)	(III)	(IV)	(I)	(II)	(III)
Home Health Care	3.8**	4.5**	2.9**	7.5**	1.7	2.2	1.7
	(2.0, 5.6)	*(2.8, 6.2)*	*(1.5, 4.4)*	*(2.5, 12.5)*	*(-2.5, 5.8)*	*(-1.9, 6.2)*	*(-2.3, 5.7)*
Nursing Home Care	2.1**	2.1**	1.6**	1.4	-1.1	-0.1	0.2
	(0.9, 3.3)	*(0.8, 3.3)*	*(3.9, 2.8)*	*(-2.1, 4.9)*	*(-3.7, 1.5)*	*(-2.8, 2.6)*	*(-1.9, 2.3)*
Any Formal Long-Term Care	4.4**	5.1**	3.6**	7.3*	1.7	2.8	2.3
	(2.8, 6.1)	*(3.3, 7.0)*	*(1.9, 5.3)*	*(1.8, 12.8)*	*(-2.7, 6.1)*	*(-1.61, 7.3)*	*(-1.8, 6.5)*
State Fixed Effect	X			X	X		
Person Fixed Effect		X				X	
Person Random Effect			X				X
Within-State Comparison Group				X			

**P≤0.01; All models contain individual level controls (restricted to time-varying individual-level controls in person fixed effects models) and year fixed effects

© 2020 Van Houtven et al. *JAMA Network Open*.

eAppendix 4. Informal Care Use

Unpaid informal long-term care may substitute for formal care use and vice-versa. To assess whether the increased LTC use following ACA expansion is offset by reductions in informal care use, we examine this outcome using the same analytic approach used for our main analyses of formal long-term care use. Informal care use was defined using a series of HRS questions that ask respondents whether they received help with any activities of daily living (ADLs) or instrumental activities of daily living (IADL). For individuals who report receiving help on any of these activities, we examine follow up questions to ascertain who provided the help. Reports of getting help from a family, friend, neighbor, or other unpaid individual were classified as having received informal care in the corresponding survey year. Table S3 present the result of our difference-in-difference analysis of this outcome, as well as two sensitivity analyses where person-level fixed effects are used instead of state fixed effects and an in-state control group (described in the following section) is used. No significant reductions in informal care were detected.

© 2020 Van Houtven et al. *JAMA Network Open*.

eTable 3. Effect of ACA-funded Medicaid Expansion on Informal Long-term Care Use

	Likely Eligible for ACA-Medicaid Expansion†		
	Change in Probability of Informal Long-Term Care Use in Expansion vs. non-Expansion States following Medicaid Expansion (percentage points)	Change in Probability of Informal Long-Term Care Use in Expansion vs. non-Expansion States following Medicaid Expansion (percentage points)	Change in Probability of Informal Long-Term Care Use in low-income vs. higher income individuals following Medicaid Expansion (percentage points)
	(95% Confidence Interval)	*(95% Confidence Interval)*	*(95% Confidence Interval)*
Informal care use	2.7	2.8	-3.6
	(-0.5, 5.9)	(0.1, 5.4)	(-10.4, 3.27)
State Fixed Effect	X		X
Person Fixed Effect		X	
Within-State Comparison Group			X

† Respondents with incomes below 138% of the federal poverty level and without Medicare coverage in 2014

© 2020 Van Houtven et al. *JAMA Network Open*.
